# Hemoglobin in Submicron Particles (HbMPs) Is Stabilized Against Oxidation

**DOI:** 10.3390/antiox13121477

**Published:** 2024-11-30

**Authors:** Pichayut Rerkshanandana, Xiaotong Zhao, Yu Xiong, Yao Chen, Axel Steffen, Saranya Chaiwaree, Chiraphat Kloypan, Axel Pruss, Radostina Georgieva, Hans Bäumler

**Affiliations:** 1 Institute of Transfusion Medicine, Charité-Universitätsmedizin Berlin, 10117 Berlin, Germany; pichayut.rerkshanandana@charite.de (P.R.); xiaotongzhao1@gmail.com (X.Z.); yu.xiong@charite.de (Y.X.); yao.chen@campus.tu-berlin.de (Y.C.); axel_steffen@gmx.de (A.S.); axel.pruss@charite.de (A.P.); radostina.georgieva@charite.de (R.G.); 2 Department of Pharmaceutical Technology and Biotechnology, Faculty of Pharmacy, Payap University, Chiang Mai 50000, Thailand; saranya_c@payap.ac.th; 3 Department of Pathology, School of Medicine, University of Phayao, Phayao 56000, Thailand; chiraphat.kl@up.ac.th; 4 Department of Medical Physics, Biophysics and Radiology, Faculty of Medicine, Trakia University, Stara Zagora 6000, Bulgaria

**Keywords:** hemoglobin, blood substitute, catalase, superoxide dismutase, methemoglobin, hydrogen peroxide, antioxidants, ascorbic acid, oxidation

## Abstract

Superoxide dismutase (SOD) and Catalase (CAT) play a crucial role as the first line of defense antioxidant enzymes in a living cell. These enzymes neutralize the superoxide anion from the autooxidation of oxyhemoglobin (Oxy-Hb) and convert hydrogen peroxides into water and molecular oxygen. In this study, we fabricated hemoglobin submicron particles (HbMPs) using the Coprecipitation Crosslinking Dissolution (CCD) technique and incorporating first-line antioxidant enzymes (CAT, SOD) and second-line antioxidant (ascorbic acid, Vit. C) to investigate a protective effect of modified HbMPs via cyclically oxygenation and deoxygenation. Thereafter, the total hemoglobin (Hb) content and Oxy-Hb content to HbMPs were determined. The results revealed that the HbMPs have a protective effect against oxidation from hydrogen peroxide and potentially neutralizing hydrogen peroxide to water over 16 times exposure cycles. No significant differences in total Hb content were found between normal HbMPs and enzyme-modified HbMPs in the absence of Vit. C. The Oxy-Hb of CAT-HbMPs showed significantly higher values than normal HbMPs. The functional Hb of normal HbMPs and enzyme-modified HbMPs was increased by 60–77% after a short time Vit. C (1:25) exposure. The co-immobilization of CAT and SOD in hemoglobin particles (CAT-SOD-HbMPs) in the presence of Vit. C provides protective effects against oxidation in cyclic Oxygenation and Deoxygenation and shows the lowest reduction of functional Hb. Our studies show that the CCD technique-modified HbMPs containing antioxidant enzymes and a reducing agent (ascorbic acid) demonstrate enhanced Hb functionality, providing protective effects and stability under oxidative conditions.

## 1. Introduction

Hemoglobin-based oxygen carriers (HBOCs) have been developed as potential alternatives to traditional blood transfusions, primarily to address significant challenges in transfusion medicine. The availability of safe and compatible donor blood remains limited, particularly in emergencies, remote areas, or during mass casualty events. Additionally, concerns about infectious disease transmission, immunogenic reactions, and the short shelf life of stored blood are ongoing issues that limit the reliability of blood supplies worldwide [1]. HBOCs aim to bridge this gap by offering a shelf-stable, universally compatible oxygen-carrying solution that can be used in patients without the need for blood type matching or complex storage conditions [2]. These carriers offer numerous advantages over traditional blood transfusions, including enhanced storage stability, reduced risk of infectious disease transmission, and compatibility with a wide range of blood types, providing a vital resource in emergency situations and medical environments where blood availability is limited or incompatible [3,4].

Despite their potential, the development of HBOCs has been fraught with challenges, particularly related to oxidative stress and hemoglobin instability. Free hemoglobin, when not encapsulated within red blood cells, can undergo rapid auto-oxidation, leading to the production of reactive oxygen species (ROS) and methemoglobin (MetHb or ferric Hb(Fe^3+^)), which impairs the oxygen-carrying capacity of the solution [5,6]. The accumulation of ROS not only reduces the efficacy of HBOCs but also poses a risk of cellular damage and inflammation, which has limited their clinical application. To overcome these issues, researchers have explored methods to enhance the antioxidant properties of HBOCs to stabilize hemoglobin and minimize oxidative damage.

Numerous strategies have demonstrated highly promising results in stabilizing tetrameric hemoglobin, such as crosslinking [7], encapsulation [8,9], and polymerization [7,10]. By stabilizing hemoglobin with these strategies, oxygen delivery in blood transfusions and other circumstances with normal partial oxygen pressures in the body was enhanced. The majority of these methods merely stabilize the alpha and beta tetramers of hemoglobin but provide no protection against free radicals, including hydroxyl and superoxide anion radicals [5] and autooxidation [11]. High concentrations of antioxidant enzymes like Superoxide dismutase (SOD) and Catalase (CAT) inside the RBCs aid in protecting hemoglobin from free radical damage [12].

One approach to enhance the stability of HBOCs is the incorporation of antioxidant enzymes such as CAT and SOD into the carrier solution. CAT catalyzes the decomposition of hydrogen peroxide (H_2_O_2_), a potent ROS, into water and oxygen, thereby preventing oxidative damage to hemoglobin [13]. SOD plays a complementary role by converting superoxide radicals, another form of ROS, into H_2_O_2_, which can then be further broken down by CAT [14]. Together, these enzymes form a synergistic antioxidant system that can reduce oxidative stress, preserve hemoglobin function, and mitigate the formation of MetHb [15,16]. Incorporating CAT and SOD into HBOCs thus offers a promising strategy for improving their safety and performance.

In addition to enzymatic antioxidants, non-enzymatic antioxidants such as ascorbic acid (Vit. C) have been shown to enhance the stability of hemoglobin by reducing oxidative stress. Vit. C is a powerful reducing agent that can prevent the oxidation of hemoglobin to MetHb by directly reducing the ferric iron in MetHb back to its functional ferrous state [17]. However, the concentration of Vit. C used is critical, as high concentrations can have pro-oxidant effects. At elevated levels, Vit. C can participate in redox cycling reactions that generate reactive oxygen species, potentially leading to oxidative damage and destruction of the heme group in hemoglobin [18,19]. This occurs because high doses of Vit. C can reduce ferric iron back to ferrous iron, but this process can also facilitate the formation of hydroxyl radicals through Fenton-type reactions, which are harmful to hemoglobin’s heme structure [18,19].

Therefore, to maximize the beneficial antioxidant effects of Vit. C while avoiding its potential pro-oxidant risks, it is important to use it at lower concentrations. Low concentrations are sufficient to reduce MetHb and mitigate oxidative stress without provoking the adverse effects associated with high levels of Vit. C. The combined use of enzymatic antioxidants like SOD and CAT, along with carefully controlled amounts of Vit. C may significantly enhance the stability and safety of HBOCs by reducing ROS levels and preserving hemoglobin functionality [6,19].

Recently, a new type of hemoglobin microparticles (HbMPs) has been produced and proposed by our group. The three crucial steps of the preparation process were published under the abbreviation CCD-technique (Co-precipitation, Crosslinking and Dissolution) [20,21,22]. In the first step, two salt solutions (e.g., manganese chloride and sodium carbonate) containing hemoglobin are mixed and form the inorganic template (co-precipitation). After that, the proteins within the template are crosslinked using glutaraldehyde (GA) or another crosslinker. Finally, the inorganic template was dissolved using EDTA, resulting in pure protein microparticles [21,23,24]. This technique provides a protein biopolymer particle with a promising result in low polydispersity, which leads to these particles having narrow size distribution, high oxygen affinity, and high protein entrapment [21,22,24].

In this study, bovine hemoglobin solution was used as the initial material to fabricate HbMPs. Cyclic oxygenation and deoxygenation experiments were performed to investigate the stability of hemoglobin in HbMPs compared with pure hemoglobin solutions. Additionally, the enzymes SOD and CAT were incorporated into the HbMPs, including their mixtures. The stabilizing effect of these enzymes was investigated in the presence and absence of Vit. C, with the goal of enhancing the antioxidant properties of HbMPs and reducing MetHb content. In this study, we tested CAT and SOD due to their specific actions against superoxide radicals and H_2_O_2_, common ROS in oxidative environments. We confirmed the functional activity of SOD and CAT post-fabrication, as measured through ROS-neutralization assays. Results indicated that the enzymes retained high activity, effectively mitigating oxidative stress in HbMPs even after multiple oxygenation and deoxygenation cycles, underscoring the stability of the preparation.

## 2. Materials and Methods

### 2.1. Material

Catalase from bovine liver (CAT), superoxide dismutase from bovine erythrocyte (SOD), l-ascorbic acid, glutaraldehyde (GA), manganese chloride tetra-hydrate (MnCl_2_·4H_2_O), sodium carbonate (Na_2_CO_3_), sodium bicarbonate (NaHCO_3_), potassium hexacyanoferrate (III) (K_3_[Fe(CN)_6_]), phosphate-buffered saline (PBS) pH 7.4, sodium nitrite (NaNO_2_), Triton-X-100 and sodium borohydride (NaBH_4_) were purchased from Sigma-Aldrich (Munich, Germany). Cobalt (II) nitrate hexahydrate (Co(NO_3_)_2_·6H_2_O) was purchased from Fluka (Seelze, Germany). Ethylene diamine tetra-acetic acid (EDTA) was purchased from Fluka (Buchs, Switzerland). Ampuwa^®^ (aqua ad injectable) and sterile 0.9% NaCl solution were purchased from Fresenius Kabi Deutschland GmbH (Bad Homburg, Germany). NaOH and Pronase^®^ were purchased from Carl Roth GmbH (Karlsruhe, Germany). Human serum albumin (HSA) solution 20% was purchased from Grifols Deutschland GmbH (Frankfurt a.M., Germany). Nitrogen gas was purchased from Linde (Berlin, Germany). Bovine hemoglobin solutions were provided by Biophyll GmbH (Dietersburg, Germany). The total Hb content (cHb) of the bovine Hb solution was measured by ABL700 (Radiometer^®^, Copenhagen, Denmark), adjusted to 50 mg/mL with 0.9% NaCl as a stock Hb solution and kept at 4 °C until needed. Methemoglobin (MetHb) solutions were obtained by oxidizing Fe^2+^ of Hemoglobin (Hb) to Fe^3+^ using sodium nitrite (NaNO_2_) [25,26]. Briefly, 5 mg/mL Hb solutions were incubated with 5 mM NaNO_2_ at room temperature (22 °C) for 30 min under gentle mixing to obtain the MetHb solution. The percentage of MetHb was measured by ABL700, and thereafter, the solutions were kept at 4 °C.

### 2.2. Particle Preparation

#### 2.2.1. Fabrication of Hemoglobin Submicron Particles (HbMPs)

HbMPs were produced using a method based on the co-precipitation, crosslinking, dissolution (CCD) technique, as described previously [23,24]. A total of 5 mg/mL Hb in 0.125 M MnCl_2_ solution was quickly mixed with 0.125 M Na_2_CO_3_ for 30 s at room temperature (co-precipitation), thereafter centrifuged and the supernatant removed. The sediment was washed twice in distilled water (3000× *g*, 3 min, room temperature) and re-suspended in a 0.9% NaCl solution. Incubation with 0.02% GA solution for 1 h at room temperature is the step to crosslink the hemoglobin in the MnCO_3_ template. Thereafter, the MnCO_3_ of the templates was dissoluted with 0.5 M EDTA (pH 7.4) for 30 min. The obtained HbMPs were washed 3 times with 0.9% NaCl solution containing 0.2 g/dL HSA (10,000× *g*, 10 min, 4 °C) and re-suspended in 0.9% NaCl solution. For further use, these particles were stored at 4 °C.

#### 2.2.2. Fabrication of Enzyme Incorporating HbMPs

**Figure 1:**
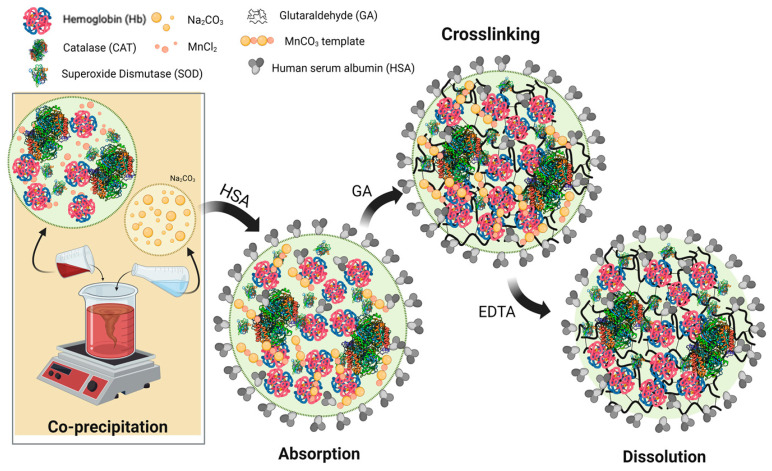
Schematic of the fabrication of hemoglobin submicron particles (HbMPs) containing enzymes (Catalase—CAT, Superoxide Dismutase—SOD, Mixture of CAT-SOD) through a Co-precipitation–Crosslinking–Dissolution (CCD) technique.

The fabrication procedure for HbMPs containing different enzymes was performed as described in Section 2.2.1, but 1 mg/mL solutions of CAT or SOD or both were added to the Hb solutions with CAT:SOD ratios like in human blood 1:16 [27] before co-precipitation was performed (Figure 1). The HbMPs with incorporated enzymes are referred to as CAT-HbMPs, SOD-HbMPs, and CAT-SOD-HbMPs, respectively.

### 2.3. Characterization of HbMPs

#### 2.3.1. Size, Zeta Potential and Morphology

The size, zeta potential and polydispersity index (PDI) were measured with Zetasizer Nano ZS (Malvern Instruments Ltd., Malvern, UK) at 25 °C. All measurements were made in triplicate (average from 12 runtimes per measurement). All particle types were suspended in 10 mM NaCl for both measurements (Size and Zeta potential). The conductivity was measured during the measurement of Zeta potential. The polydispersity index (PDI) value was obtained after a complete run on size measurement.

#### 2.3.2. Determination of Oxygen Carrying Capacity of HbMPs

It is necessary to quantify the part of Hb that can carry and release oxygen, the so-called Oxy-Hb or functional Hb. In contrast, MetHb cannot bind or carry oxygen [28,29]. The transportation of oxygen to the tissues is reduced as MetHb levels rise [30,31]. The Oxy-Hb content of all HbMPs types was measured using the ferricyanide technique [32]. Briefly, 2% (*v*/*v*) particle suspension was added to a 2 mL glass vial containing a magnetic stirrer. The concentration of dissolved oxygen was measured and recorded using a portable fiber optic needle-type oxygen sensor (Needle-Type Oxygen Microsensor NTH-PSt7, PreSens-Precision Sensing GmbH, Regensburg, Germany) connected to a portable oxygen meter with data logging (Microx 4, PreSens-Precision Sensing GmbH, Regensburg, Germany). After reaching a stable baseline (starting point), potassium ferricyanide (K_3_[Fe(CN)_6_]) (100 mg/mL) was added to remove the oxygen from the oxygenated hemoglobin, transferring it to MetHb. The dissolved oxygen was monitored until it reached a constant value. The oxygen bound to hemoglobin in the particles is calculated by subtracting the end $pO_{2}$ value from the starting $pO_{2}$ value. The following formula, known as Henry’s Law, was used to convert the measured difference in $pO_{2}$ into the concentration of released oxygen ($cO_{2}$).

(1) $$cO_{2}=aO_{2}\times pO_{2}$$

where $aO_{2}$ represents the solubility coefficient of oxygen in the blood (0.031 mL (O_2_)/mmHg (O_2_)/L blood). The ${pO}_{2}$ is the partial pressure of oxygen. The concentration of the Oxy-Hb (c(Oxy-Hb)) was calculated as follows:

(2) $$c\left(Oxy-Hb\right)=\frac{mO_{2}}{\rho O_{2}\times Hb_{s}}$$

where $\rho O_{2}\;$ = 1.43 g/L is the density of $O_{2}$, and $Hb_{s}$ is the saturated Oxy-Hb content which equals 1.34 mL of $O_{2}$ per gram of Hb. The mass of released oxygen ($mO_{2}$) was transformed from $pO_{2}\;$ by subtracting the end $mO_{2}$ value from the starting $mO_{2}$ value, which was multiplied by the volume of samples in mL unit.

#### 2.3.3. Determination of Total Hb Content

A modified alkaline hematin detergent technique (AHD method) was used to assess the total Hb concentration of modified HbMPs [22,33]. In brief, to 1 mL of 2% (*v*/*v*) particle suspensions, Pronase^®^ (10 mg/mL) was added to achieve a final concentration of 0.5 mg/mL and the suspensions were then incubated at 45 °C for 30 min. The AHD reagent (25 mg/mL Triton X-100 in 0.1 M NaOH) was then added in a volume ratio of 1:1, and the solutions were then gently mixed and incubated at room temperature for 15 min. The absorbance of all samples was measured at a wavelength of 574 nm using a UV-vis spectrophotometer (Cary 60 UV-Vis Spectrophotometer, Agilent, Waldbronn, Germany), and the total Hb content was calculated using an average of five individual measurements.

### 2.4. Stability of HbMPs Against Oxidizing Agents

Biological tissues have the ability to protect themselves against hydrogen peroxide (H_2_O_2_) as an oxidizing agent by means of catalase. Therefore, it should be proven whether HbMPs can defend themselves against H_2_O_2_. Hydrogen peroxide can oxidize cobalt (II) to cobalt (III), which forms the colored carbonate-cobaltate (III) complex ([Co(CO_3_)_3_]Co) in the presence of bicarbonate as shown in the reaction (3). At 440 and 640 nm, this complex exhibits two distinct absorption peaks. The obtained absorbance value is proportional to hydrogen peroxide concentration [34]. This method was applied to quantify the remaining H_2_O_2_ in the HbMPs suspensions after adding it to the suspension.

2[Co(H_2_O)_6_]^2+^ + 4HCO_3_^−^ + H_2_O_2_ → [Co(CO_3_)_3_]Co + CO_2_ + 15H_2_O (3)

Briefly, 2% (*v*/*v*) HbMPs were quickly mixed with H_2_O_2_ (2–8 mM). Then, the mixture was centrifuged at 5000× *g*, for 5 min at room temperature. The supernatants were collected and added to the working solution, containing cobalt (II) and bicarbonate solution, and incubated for 10 min in the dark [34]. Finally, the samples were measured at 440 and 640 nm spectrophotometrically. The absorbance values obtained are proportional to the free H_2_O_2_ in the supernatant after the oxidation process in the presence of HbMPs.

To evaluate the stability of HbMPs, the procedure was repeated by centrifuging, washing, and resuspending the samples in 0.9% NaCl. This process was then cycled by re-incubating the samples with H_2_O_2_ (2–8 mM) and performing spectrophotometric measurements after each cycle until an increase in absorbance was observed.

### 2.5. Enhancing Functional Hb of HbMPs

#### 2.5.1. Optimal Conditions for Incorporating Vit. C with HbMPs

Vit. C with different concentrations (molar ratios to Hb of 1:250, 1:90, 1:50 and 1:25) was incubated with Hb up to 72 h at pH 7.4 and 4 °C. At the time points 0, 1, 2, 4, 24, 48 and 72 h after incubation, the MetHb and Hb content were measured by ABL700. The percentages of MetHb reduction and Hb loss were calculated in comparison to values at time point 0.

#### 2.5.2. Effect of Enzymes (CAT, SOD, CAT-SOD) and Vit. C on HbMPs

Vit. C was used to prove if it can be used as a protective agent of Hb against oxidation in all types of HbMPs. In brief, Vit. C (1 mM) was gently mixed with 2% (*v*/*v*) HbMPs for 30 min at room temperature. Then, the particles were washed twice with 0.9% NaCl (6000× *g*, 10 min, 4 °C). Thereafter, the functional Hb of HbMPs was determined using the modified AHD method and ferricyanide technique (Section 2.3.2 and Section 2.3.3).

#### 2.5.3. Cyclic Oxygenation and Deoxygenation of HbMPs and HbMPs with Incorporated-Enzymes (CAT, SOD, CAT-SOD)

HbMPs, CAT-HbMPs, SOD-HbMPs, and CAT-SOD-HbMPs, after treatment with Vit. C (Section 2.5.2) underwent cyclic oxygenation and deoxygenation to evaluate their oxygen-binding capacity over multiple cycles. A 4 mL suspension of each HbMP type was placed in airtight glass vials with moderate stirring. A Needle-Type Oxygen Microsensor was inserted through a rubber-sealed lid to continuously monitor partial oxygen pressure (pO_2_) throughout the entire process.

The deoxygenation process began by supplying wet nitrogen gas to the system for 2 min at 18 °C, reducing the oxygen content in the HbMPs suspension. After deoxygenation, oxygenation was performed by introducing normal wet air for 2 min using a portable air vacuum pump (VWR International, Leuven, Belgium). This cycle of nitrogen-induced deoxygenation and air-induced oxygenation was repeated 10 times, with pO_2_ monitored during each phase.

The experiment ended after the 10th oxygenation cycle, and the percentage of functional Hb in the HbMPs was determined by using the modified AHD method and ferricyanide technique (Section 2.3.2 and Section 2.3.3). This approach allowed for an assessment of the stability, functionality, and oxygen-binding capacity of HbMPs and enzyme-modified HbMPs (CAT, SOD, CAT-SOD) under cyclic oxidative stress conditions.

### 2.6. Statistical Analysis

All experimental data were expressed as the mean ± standard deviation (SD) of at least three independent experiments. The statistical significance of differences between groups was assessed using a one-way analysis of variance (ANOVA) followed by Tukey’s multiple comparisons test. This approach allowed for the comparison of multiple groups simultaneously, providing insight into differences across various experimental conditions.

For all statistical tests, a significance level of *p* < 0.05 was considered to indicate statistical significance. Asterisks (*) in the figures denote statistically significant differences compared to the control hemoglobin microparticles (HbMPs). Statistical analyses were performed using [GraphPad Prism Version 5.0], ensuring rigor in the evaluation of the experimental results.

## 3. Results and Discussion

### 3.1. Fabrication and Characterization of HbMPs Containing Enzymes (CAT, SOD, CAT-SOD)

HbMPs containing enzymes (CAT, SOD, CAT-SOD) were fabricated using the MnCO_3_ template. As described in our group’s previous [21], the MnCO_3_ template entraps proteins with higher efficacy than the CaCO_3_ template due to the high protein affinity of Mn^2+^ [35,36].

Glutaraldehyde was used to crosslink hemoglobin and enzymes through an abundance of amino groups [37,38], forming the hemoglobin-enzymes complex. The crosslinking with GA showed promising low immunogenicity and increased oxygen affinity to impede premature oxygen release and vasoconstriction caused by an autoregulation process [21,39,40].

#### 3.1.1. Size and Zeta Potential

The hydrodynamic diameter of all HbMPs types in NaCl solution was measured by dynamic light scattering and showed an average size of HbMPs, CAT-HbMPs, SOD-HbMPs and CAT-SOD-HbMPs in a range between 600 and 800 nm (Table 1). SOD is a 32 kDa homodimeric β-barrel protein containing an intramolecular disulfide bond and a binuclear Cu/Zn site responsible for catalyzing the disproportionation of superoxide to hydrogen peroxide and dioxygen [41,42]. This SOD has a half molecular weight of tetrameric protein hemoglobin, 32.5 kDa. The molecular mass of CAT is about 240 kDa, containing four identical subunits of 62 kDa polypeptide chains [43]. Obviously, the sizes of enzymes incorporating HbMPs are not significantly different, and therefore, the different molecular weight of the enzymes has no influence on the particle formation process. The average polydispersity index (PDI) value of all types was lower than 0.2, demonstrating a homogenous size distribution of all HbMP types as well as a low level of aggregation, which is acceptable in drug delivery applications [44].

The zeta potential values for all HbMPs types (Table 1), measured in 10 mM NaCl solutions with conductivity ranging from 1.31 to 1.38 mS/cm, were uniformly negative, averaging approximately –30 mV. This pronounced negative zeta potential likely results from human serum albumin (HSA) adsorption occurring during washing steps after the dissolution process. No statistically significant differences in zeta potential were observed among the HbMP types (*p* < 0.05).

#### 3.1.2. Total Hb Content and Oxygen Carrying Capacity

The Hb content in all HbMP types was determined using the modified pronase-AHD technique. The pronase was used to break down the peptide bonds in proteins and convert the particle suspension into a clear solution. The AHD reagent (25 mg/mL Triton X-100 in 0.1 M NaOH) converts all the different Hb variants into the complex of alkaline hematin and a non-ionic detergent and enables the photometric determination of the Hb concentration. The entrapped Hb by MnCO_3_ template of all HbMPs types was ~54 fg Hb/particle, which was in the range previously reported by our group [20,22,24,40].

**Figure 2:**
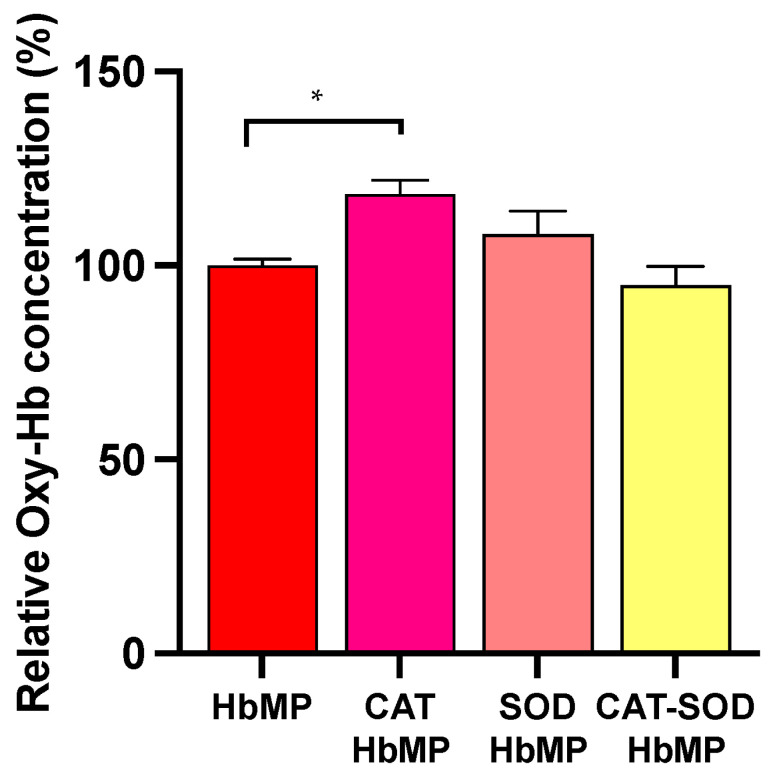
Relative oxygenated Hb (Oxy-Hb) concentration of all HbMP types. Data are presented as a mean ± SD (*n* = 3). One-way ANOVA and Turkey’s multiple comparisons were utilized to calculate the data. The asterisk indicates data have a statistically significant difference compared to HbMPs (* *p* < 0.05).

The ferricyanide method was applied to quantify the Oxy-Hb concentration representing the oxygen-carrying capacity of HbMPs as a simple and inexpensive method. The highly reactive cyanide ion in ferricyanide liberates oxygen from oxyhemoglobin and transforms itself to MetHb [32]. The release of the oxygen from ferricyanide’s reaction in HbMPs suspension, as described in Section 2.3.2, was then proportional to the amount of oxygen bound to Oxy-Hb, representing the oxygen binding capacity of HbMPs. Of all types of HbMPs, only CAT-HbMPs show a slightly significant difference in relative Oxy-Hb content from normal HbMPs in Figure 2. On the contrary, CAT-SOD-HbMPs show no significant difference from normal HbMPs.

Interestingly, SOD-HbMPs slightly enhance the Oxy-Hb content compared to normal HbMPs, but with non-significant differences. This is probably caused by the antioxidant property of conjugated enzymes enhancing the ability of our HbMPs to maintain a partial oxygen level and to protect the tissue from hypoxia damage [45,46]. The percentage of Oxy-Hb is used to imply oxygen binding capacity in HbMPs.

### 3.2. Stability of HbMPs Against Oxidizing Agents

**Figure 3:**
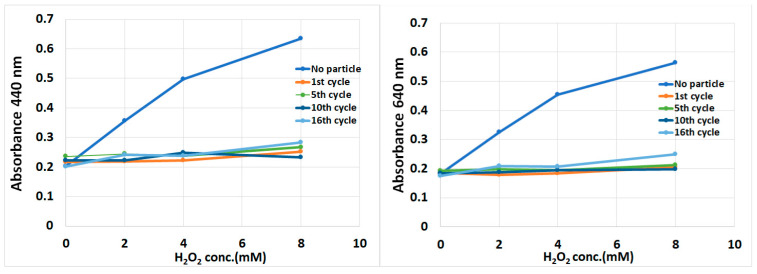
The absorbance of carbonate-cobaltate (III) complex ([Co(CO_3_)_3_]Co) at 440 nm (**Left**) and 640 nm (**Right**) in the presence of hydrogen peroxide (H_2_O_2_). Higher absorbance indicates a higher concentration of free/reactive hydrogen peroxide in the suspension. The experiment was cyclically performed 16 times on the same day with identical samples.

In this experiment, we point out that hemoglobin in HbMPs is protected against oxidizing agents. We added H_2_O_2_ (oxidizing agent) to the HbMPs suspensions and measured free-H_2_O_2_ in the supernatant using the carbonate-cobaltate (III) complex ([Co(CO_3_)_3_]Co) method. The decrease of H_2_O_2_ demonstrates the catalytic function of Hb in the HbMPs. Figure 3 shows the absorption of carbonate-cobaltate complex (III) in HbMP suspensions treated with different concentrations of H_2_O_2_ (2–8 mM) at 440 and 640 nm. The absorbance values correspond to the amount of free H_2_O_2_ in the supernatant and show the highest absorbance value in the control solution (without HbMPs). On the other hand, normal HbMPs (1 mg/mL), which were treated with H_2_O_2_ of different concentrations, showed no change in absorbance value at the first cycle and no significant difference after the 16th cycle. This result demonstrates that free-H_2_O_2_ was undergoing some reaction, which might be within the heme of hemoglobin [47].

Hydrogen peroxide (H_2_O_2_), a reactive oxygen species (ROS), plays a crucial role in oxidative stress within biological systems, including hemoglobin-based oxygen carriers (HBOCs). When free hydrogen peroxide is present, it can participate in redox reactions with hemoglobin, particularly with the heme group, which contains an iron center capable of undergoing oxidation and reduction cycles [48].

The heme iron in hemoglobin can engage in Fenton-like reactions where hydrogen peroxide acts as an oxidizing agent. In this process, ferrous Hb(Fe^2+^) reacts with H_2_O_2_, initially forming a complex Hb(Fe^2+^)(H_2_O_2_) that further converts to ferryl-Hb peroxide (Fe^4+^=O) or breaks down to ferric Hb(Fe^3+^) (MetHb) and a hydroxyl radical (•OH) [49,50]. The redox cycling between ferryl-/ferric and ferrous states can aid in the reduction of hydrogen peroxide within the solution as part of the heme’s ability to process ROS [6,19]. Through these redox reactions, some of the free hydrogen peroxide is broken down, potentially mitigating its accumulation and reducing its cytotoxic effects.

However, while these redox reactions can reduce the levels of free hydrogen peroxide, they also pose risks to the stability of hemoglobin. The formation of reactive intermediates, such as ferryl hemoglobin and hydroxyl radicals, can lead to oxidative damage to the heme and globin chains, contributing to the formation of MetHb (Hb(Fe^3+^)), which is incapable of binding oxygen [6,15,16,47]. In addition, the reactive nature of hydroxyl radicals can lead to lipid peroxidation, protein oxidation, and cellular damage, particularly in the absence of sufficient antioxidant defenses.

To address this, HBOCs often incorporate antioxidant enzymes such as catalase (CAT) and superoxide dismutase (SOD) to further reduce ROS and protect against oxidative damage [51]. Catalase plays a pivotal role by converting hydrogen peroxide into water and oxygen, thus preventing its accumulation and the associated oxidative stress [13,14,15,16]. This enzyme effectively neutralizes hydrogen peroxide before it can participate in deleterious redox reactions within the heme group.

The redox reactions within hemoglobin may reduce the concentration of free hydrogen peroxide in solution, but the presence of external antioxidants like catalase and Vit. C is critical to maintain balance and prevent excessive oxidative stress. Vit. C, for example, can directly reduce MetHb back to its functional ferrous state (Fe^2+^), further mitigating the oxidative effects of hydrogen peroxide [17]. However, care must be taken with antioxidant levels, as excess Vit. C could act as a pro-oxidant in the presence of high levels of hydrogen peroxide or free iron, accelerating oxidative damage instead of preventing it [6,17,19,51].

### 3.3. Enhancing Functional Hb of HbMPs

#### 3.3.1. Optimal Conditions for Incorporating Vit. C with HbMPs

**Figure 4:**
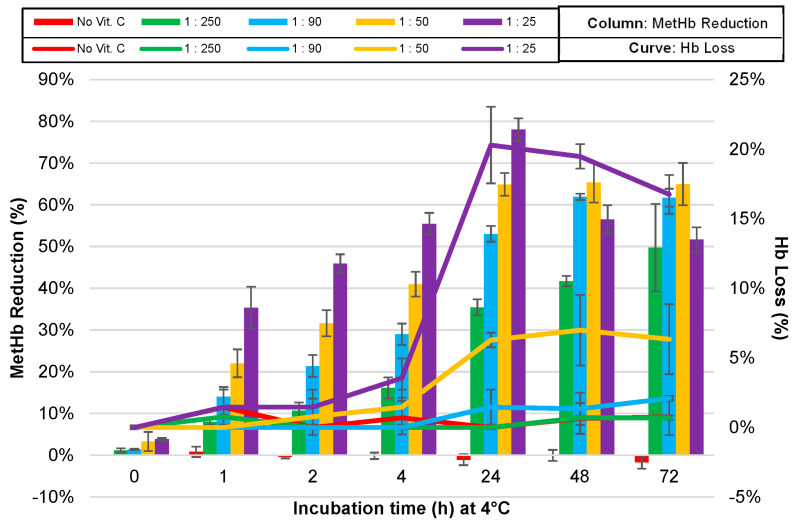
Methemoglobin (MetHb) reduction (column) and hemoglobin (Hb) loss (curve) over time during incubation of Hb solution with varying concentrations of Vit. C in molar ratio of Vit. C to Hb at 4 °C and pH 7.4. Data are presented as a mean ± SD (*n* = 3).

The balance between effective MetHb reduction and Hb stability observed in this study is consistent with previous research, which highlights the importance of controlling Vit. C concentrations to minimize oxidative damage to Hb [6,47]. Our findings (Figure 4) on MetHb reduction demonstrated that a 1:25 molar ratio of Vit. C to Hb exhibited the highest efficacy in terms of MetHb reduction, reaching approximately 80% after 24 h, indicating that this concentration is highly effective at restoring Hb functionality by reducing MetHb.

However, this was accompanied by a significant Hb loss, approximately 20% after 24 h, suggesting that prolonged exposure at this concentration compromises Hb stability through oxidative degradation. Lower concentrations, such as at ratios 1:90 and 1:250, resulted in minimal MetHb reduction (below 30%) while keeping Hb loss under 5%. Their limited impact on MetHb suggests that these concentrations are insufficient for practical therapeutic use.

**Figure 5:**
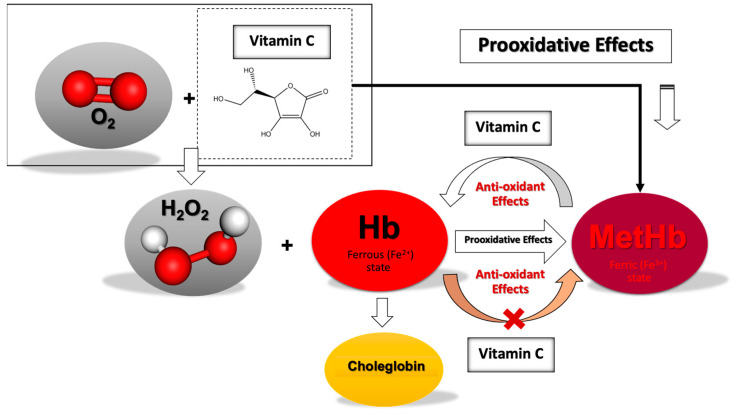
Interaction of Vit. C with Hemoglobin and its Dual Role in Antioxidant and Pro-oxidative Effects.

The efficacy of Vit. C in reducing MetHb and its effect on Hb stability appears to be concentration-dependent. Vit. C can act both as an antioxidant and a pro-oxidant, depending on the context and concentration. At a higher concentration ratio (1:25), Vit. C effectively reduces MetHb (Fe^3+^) to Hb (Fe^2+^) but simultaneously promotes pro-oxidative effects that accelerate Hb degradation, leading to the formation of choleglobin and other byproducts [52,53] (Figure 5). Vit. C at a ratio of 1:25 offers the most rapid and significant reduction of MetHb, making it a potent reducing agent in the short term, and a suitable option for incorporation into HbMPs for therapeutic purposes. However, the downside is the associated Hb degradation, as indicated by the significant Hb loss observed after 24 h. This degradation could be demonstrated through the formation of reactive oxygen species (ROS) and oxidative intermediates, such as ferryl heme, which can damage Hb and increase Hb loss under pro-oxidative conditions under the higher Vit. C concentration [15,16,18,19].

Vit. C at the molar ratio to Hb of 1:50 demonstrated a more balanced outcome, with a 65% MetHb reduction at 24 h and a more moderate Hb loss of approximately 7%. This concentration provides a compromise between effective MetHb reduction and maintaining Hb integrity, reducing the risk of excessive degradation. Based on the findings, a Vit. C to hemoglobin ratio of 1:50 is optimal for incorporation into bovine Hb solutions. This ratio strikes a critical balance between efficient MetHb reduction and minimal hemoglobin degradation, ensuring long-term stability and preserving Hb functionality. The moderate concentration of Vit. C at 1:50 effectively reduces oxidative stress while preventing excessive pro-oxidant effects, making it particularly suited for therapeutic applications where sustained Hb activity is required over extended periods.

On the other hand, the 1:25 ratio, while demonstrating more rapid MetHb reduction, is primarily beneficial for short-term applications. It offers enhanced antioxidant action in the initial phases, with minimal hemoglobin loss (<2%), making it ideal for processes requiring quick reactions with HbMPs. However, the higher concentration increases the risk of oxidative degradation over prolonged exposure, which may compromise Hb’s integrity in long-term therapeutic strategies.

#### 3.3.2. Effect of Enzymes (CAT, SOD, CAT-SOD) and Vit. C on HbMPs

The Hb in the erythrocyte is protected against oxidation due to the reductase systems with superoxide dismutase (SOD), catalase, and MetHb reductase [53,54,55,56]. The erythrocyte plays an important role in transporting oxygen through the circulatory system and delivering oxygen to the tissues. It contains a significant portion of the body’s required oxygen and comes into close contact with all body tissues. Due to the proximity of erythrocytes in capillaries to body tissues, it is possible for compounds produced by secondary reactions involving Hb to be transferred to the tissues and thus affect the organism’s proper functioning. One of these secondary reactions of Hb is autoxidation [47,57].

Oxyhemoglobin (Oxy-Hb) and MetHb are both involved in a redox cycle triggered by the autooxidation of Oxy-Hb and catalytic reduction from NADH-Cytochrome b5 Oxido-Reductase (CYB5R) [58]. MetHb and superoxide anion is produced from the autooxidation of Oxy-Hb [47,59]. A reduction in pH or the displacement of dioxygen from Oxy-Hb by anionic ligand (Cl^−^, F^−^, CN^−^ or others) can also promote superoxide anion, which subsequently converted to H_2_O_2_ either through self-dismutation or catalytic dismutation by SOD [60].

**Figure 6:**
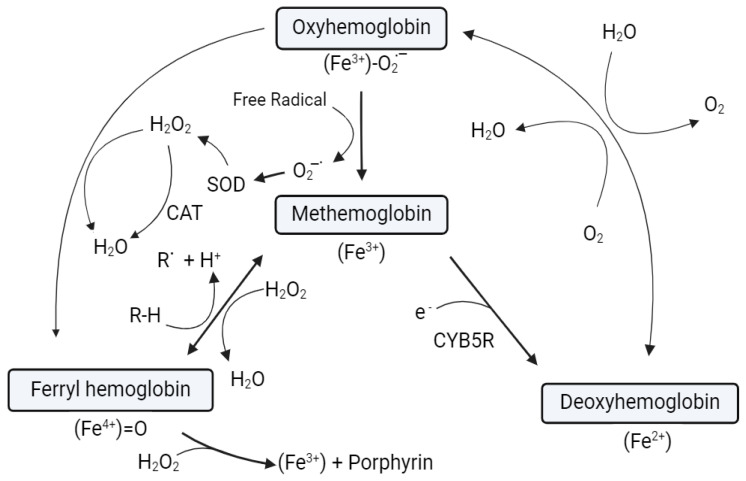
Summarizes the influence of hydrogen peroxide on Hb described in the literature [47,61].

Two additional mechanisms involving Oxy-Hb are activated by the H_2_O_2_ released during superoxide dismutation. The concentration of H_2_O_2_ in red blood cells is approximately 2 × 10^−10^ M at steady-state concentration [58]. Reacting Oxy-Hb with H_2_O_2_ produces the ferryl Hb [(Fe^4+^)=O] species, which, when combined with a second H_2_O_2_ molecule, generates MetHb and superoxide in Figure 6. Moreover, excess superoxide can cause the porphyrin to break down and release iron into the cell [52].

MetHb, the core of the Hb redox chain, can be reduced or oxidized to deoxyhemoglobin via NADH-Cytochrome b5 Oxidoreductase (CYB5R) or ferryl Hb radical cation with H_2_O_2_. Oxygenation of deoxyhemoglobin can reversibly generate Oxy-Hb. A second H_2_O_2_ can also eliminate the porphyrin and turn Oxy-Hb into ferryl Hb. Ferryl Hb may also oxidize another reduced species to become MetHb. Oxy-Hb may also spontaneously produce ROS, including superoxide, which is converted to H_2_O_2_ by SOD. Reduced agents and reductase with antioxidant capabilities were commonly utilized to prevent MetHb oxidation and free radical scavenging. Vit. C is a well-known reductant and has been well-established in a variety of biological systems in vivo, including blood [62].

**Figure 7:**
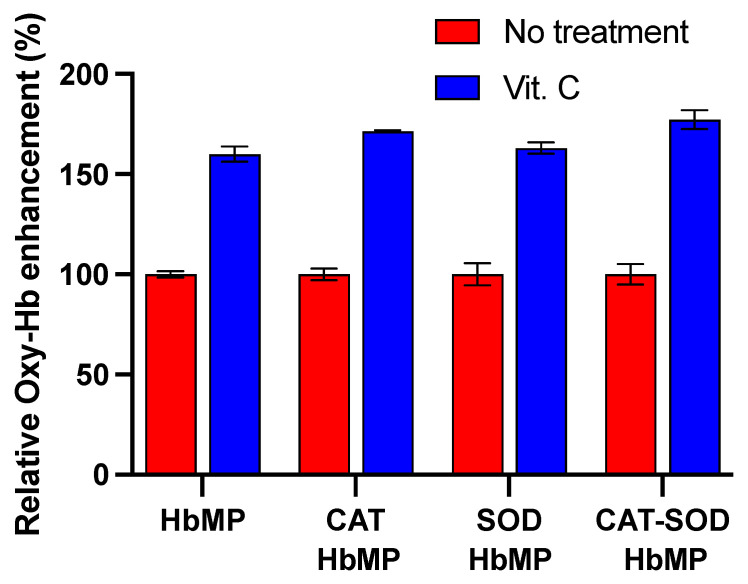
Characterization of all HbMP types shows the relative enhancement of Oxy-Hb compared between no treatments and treated with Vit. C in molar ratio of Vit. C to Hb 1:25. Data are presented as a mean ± SD (*n* = 4).

Our study explored the antioxidant effects of Vit. C on modified HbMPs. Figure 7 shows the relative Oxy-Hb enhancement of all types of HbMPs. The enhancement of Oxy-Hb content was observed at all types of HbMPs after being treated with Vit. C demonstrates that adding Vit. C on the HbMPs reduced the formation of MetHb [15,16]. These findings suggest that the antioxidant reaction of Vit. C in HbMPs influenced the improvement of the oxygen-carrying capacities of HbMPs in a range of 60–77% compared to the control group (No Vit. C treatment). Furthermore, Vit. C, in combination with CAT-HbMPs and CAT-SOD-HbMPs, shows a slight increase in the Oxy-Hb enhancing effect compared with normal HbMPs and SOD-HbMPs. This could imply that a major CAT or a combination of CAT and SOD only slightly impacts Oxy-Hb formation by Vit. C addition.

Combining Vit. C with enzymes (SOD and CAT) provides an additive antioxidant effect that surpasses the benefits of using each component alone. While Vit. C provides immediate, direct reduction of MetHb and scavenging of free radicals; the enzymes offer ongoing protection by continuously detoxifying ROS that is generated during the oxygen transport process [13,17]. This combination not only enhances the stability and oxygen-carrying capacity of the HBOCs but also reduces the risk of oxidative damage to tissues and cells.

Furthermore, the enzymatic action helps to regulate the concentration of H_2_O_2_, which can otherwise accumulate and participate in damaging redox reactions. The combined use of these antioxidants minimizes the need for high concentrations of any single antioxidant, reducing the risk of pro-oxidant effects while maintaining robust protection against oxidative stress [6,52,53].

#### 3.3.3. Cyclic Oxygenation and Deoxygenation of HbMPs and HbMPs with Incorporated-Enzymes (CAT, SOD, CAT-SOD)

**Figure 8:**
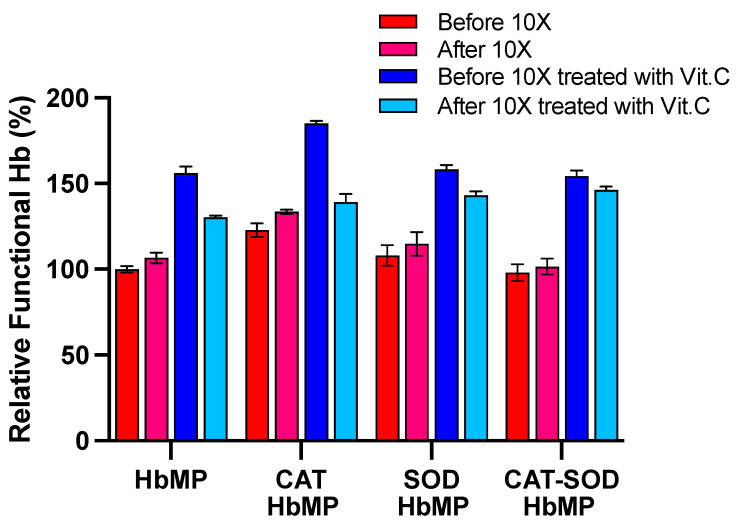
Relative functional Hb levels of all HbMP types (HbMPs, CAT-HbMPs, SOD-HbMPs, and CAT-SOD-HbMPs) before and after 10 times cyclic oxygenation and deoxygenation procedure, with and without Vit. C. Data are presented as mean ± SD (*n* = 4).

The cyclic oxygenation and deoxygenation experiments were performed to evaluate the stability of HbMPs incorporating enzymes, including CAT, SOD and Vit. C. The result shows the relative functional Hb before and after 10 cycles of oxygenation and deoxygenation for different HbMP formulations in the presence and absence of Vit. C (Figure 8). Without Vit. C. the relative functional Hb did not change substantially after cyclic oxygenation in the presence of Vit. C, the relative functional Hb decreased in all formulations after cyclic oxygenation. Notably, SOD-HbMPs and CAT-SOD-HbMPs in the presence of Vit. C displayed the highest retention of functional Hb after 10 cycles. CAT-HbMPs showed the most significant increase in functional Hb after treatment with Vit. C and before cyclic oxygenation, however, they exhibited the highest reduction after 10 cycles in the presence of Vit. C.

The results reveal significant insights into the roles of Vit. C, CAT, and SOD in preserving the functionality of HbMPs during cyclic oxygenation and deoxygenation. Vit. C, known for its antioxidant properties, initially reduces MetHb back to its functional form. This reduction is evident in all HbMP formulations before cyclic treatment, as Vit. C effectively prevents initial oxidation, thereby enhancing functional Hb levels, while its pro-oxidant potential in the presence of free iron or H_2_O_2_ might lead to the destabilization of Hb [17,52,53]. This finding is significant as it suggests that while Vit. C can provide immediate antioxidant benefits, but its use needs to be carefully controlled to prevent adverse effects on the stability of HbMPs.

However, the effectiveness of Vit. C alone appears limited after prolonged cyclic exposure, as seen in formulations without enzymatic support, such as CAT-HbMP with Vit. C. Although CAT decomposes H_2_O_2_, without SOD to neutralize superoxide radicals, H_2_O_2_ may accumulate, leading to oxidative stress that compromises Hb stability [13,14,15,16]. CAT alone cannot provide sustained protection under cyclic oxidative conditions. Additionally, a high reduction in CAT-HbMPs could be demonstrated by an interaction of nitrite ion, selectively produced from the exposure of nitrogen dioxide (NO_2_) and CAT molecule, which leads to nitrite-induced catalase inhibition under hypoxic conditions [63].

In contrast, SOD-HbMPs and CAT-SOD-HbMPs formulations with Vit. C exhibits a more favorable outcome. SOD converts superoxide radicals to H_2_O_2_, providing a complementary action that helps catalase perform optimally by breaking down H_2_O_2_ into water and oxygen. This combined effect minimizes oxidative stress during cyclic oxygenation-deoxygenation. The CAT-SOD-HbMPs formulation with Vit. C demonstrated the lowest reduction in functional Hb after 10 cycles, underscoring the synergy between CAT and SOD. This co-immobilization not only buffers oxidative species more effectively but also prevents cumulative oxidative damage [46], maintaining Hb functionality over extended cycles.

In RBCs, the ratio of Hb:SOD:CAT by mg/mL is roughly 1:0.008:0.001 (assuming an approximate Hb concentration of 300 mg/mL), reflecting a balance where antioxidant enzymes are present in sufficient quantities to manage ROS but do not overwhelm the main function of oxygen transport. A higher ratio of Hb:SOD:CAT in CAT-SOD-HbMPs of 1:0.05:0.003 was used to enable better antioxidation. However, the optimal ratio is still to be investigated.

Dr. Chang’s group formulated polymerized Hb with SOD, CAT and carbonic anhydrase (Poly-[Hb-SOD-CAT-CA]) as soluble nanobiotherapeutic [64] to support oxygen transport and antioxidant defense, with applications in liver protection [65]. Our CAT-SOD-HbMPs emphasize stability and oxygen-carrying capacity, integrating SOD and CAT to prevent hemoglobin degradation, making them ideal for transfusion applications. Unlike soluble Poly-[Hb-SOD-CAT-CA], the significantly larger particle size of HbMPs was developed to reduce the possible hypertensive side effects of the blood substitutes [20].

Overall, these findings demonstrate that incorporating enzymatic antioxidants like SOD and CAT into HbMPs is more effective in preserving the functional stability of hemoglobin during cyclic oxygenation and deoxygenation. In contrast, the addition of Vit. C, while beneficial in some contexts, may lead to unintended oxidative effects that reduce hemoglobin stability.

## 4. Conclusions

Our data demonstrated that antioxidant enzymes could be successfully entrapped with Hb. In particular, the double enzymes incorporated HbMPs (CAT-SOD) exhibit the most promising protective effects compared with a single enzyme (CAT or SOD) uses, in agreement with Nadithe’s studies [46], reveals the protective effects of crosslinked Hb-superoxide dismutase-catalase (Hb-SOD-CAT) which possesses an antioxidant mechanism, provided significant protection on Hb against free radical stresses.

Moreover, incorporating Vit. C and antioxidant enzymes into HbMPs using technique provides a multifaceted defense against oxidative stress, enhancing the stability, functionality, and safety of the functional hemoglobin. However, it is crucial to control the concentration of Vit. C is used in this context. At high concentrations, Vit. C can paradoxically act as a pro-oxidant, particularly in the presence of free iron or excess hydrogen peroxide. Therefore, using Vit. C at a low but effective concentration provides antioxidant protection without promoting oxidative damage, a balance that improves the overall safety and efficacy of HbMPs.

This combined strategy offers significant advantages over using HbMPs alone, ensuring sustained oxygen delivery, reduced oxidative damage, and improved clinical outcomes, particularly in scenarios where oxidative stress is a major concern.

## Figures and Tables

**Table 1:** Hydrodynamic diameter and zeta-potential (measured at conductivities 1.31–1.38 mS/cm) of all HbMPs types. Measurements were performed at room temperature. Data are presented as mean ± SD (*n* = 3).

HbMP Type	Size (nm)	PDI	Zeta Potential (mV)
Normal	741 ± 12	0.18 ± 0.02	−29.6 ± 1.7
CAT-HbMP	804 ± 45	0.23 ± 0.05	−29.9 ± 1.1
SOD-HbMP	689 ± 39	0.17 ± 0.09	−29.1 ± 1.2
CAT-SOD-HbMP	761 ± 22	0.09 ± 0.05	−28.6 ± 1.7

## Data Availability

Data are available after contacting the corresponding author and stored on the central server of Charité-Universitätsmedizin Berlin.
